# Functional Neuroimaging During Asleep DBS Surgery: A Proof of Concept Study

**DOI:** 10.3389/fneur.2021.659002

**Published:** 2021-06-28

**Authors:** Francesco Sammartino, Paul Taylor, Gang Chen, Richard C. Reynolds, Daniel Glen, Vibhor Krishna

**Affiliations:** ^1^Department of Neurosurgery, The Ohio State University, Columbus, OH, United States; ^2^Scientific and Statistical Computing Core, National Institute of Mental Health, National Institutes of Health, Bethesda, MD, United States

**Keywords:** deep brain stimulation, asleep DBS, functional magnetic resonance imaging, intraoperative, feedback

## Abstract

**Object:** A real-time functional magnetic resonance imaging (fMRI) feedback during ventral intermediate nucleus (VIM) deep brain stimulation (DBS) under general anesthesia (or “asleep” DBS) does not exist. We hypothesized that it was feasible to acquire a reliable and responsive fMRI during asleep VIM DBS surgery.

**Methods:** We prospectively enrolled 10 consecutive patients who underwent asleep DBS for the treatment of medication-refractory essential tremor. Under general anesthesia, we acquired resting-state functional MRI immediately before and after the cannula insertion. Reliability was determined by a temporal signal-to-noise-ratio >100. Responsiveness was determined based on the fMRI signal change upon insertion of the cannula to the VIM.

**Results:** It was feasible to acquire reliable fMRI during asleep DBS surgery. The fMRI signal was responsive to the brain cannula insertion, revealing a reduction in the tremor network's functional connectivity, which did not reach statistical significance in the group analysis.

**Conclusions:** It is feasible to acquire a reliable and responsive fMRI signal during asleep DBS. The acquisition steps and the preprocessing pipeline developed in these experiments will be useful for future investigations to develop fMRI-based feedback for asleep DBS surgery.

## Introduction

Magnetic resonance imaging (MRI) during surgery augments the real-time visualization of the brain anatomy. Therefore, intraoperative MRI was deployed to improve the accuracy of neurosurgical procedures since the 1990's (1). The seamless integration of MRI into the surgical workflow has enabled performing deep brain stimulation (DBS) under general anesthesia (or asleep DBS) (2). The electrode placement was shown to be highly accurate, and patient outcomes in experienced centers were comparable with the conventional “awake” DBS (3, 4). In parallel to the technological advancements, asleep DBS also motivated neuroimaging research to enhance the visualization of therapeutic targets (5). While structural imaging proved adequate for some therapeutic targets, tractography has emerged as the primary technique for improved localization of the ventral intermediate nucleus (VIM) for DBS surgery (6, 7).

Besides anatomical accuracy, MRI-based physiological feedback can provide another layer of verification during asleep DBS surgery (8). Intraoperative functional MRI (fMRI) is the preferred technique for providing physiological feedback during asleep DBS (9). While the determination of safety conditions required for scanning DBS patients using high-field strength MR is an important first step (10), it remains unclear whether a high-quality fMRI signal can be acquired during asleep DBS. Specific concerns include the artifacts from surgery (i.e., blood products from the surgical incision and burr hole), the associated hardware (i.e., head immobilization pins and stereotactic frame), and the preprocessing pipelines to correct any distortions are yet to be developed. It is also unknown whether the electrode passage during DBS, without stimulation, induces changes in the fMRI signal. This determination is relevant because brain transgression with DBS electrodes, without stimulation, can induce transient but clinically detectable changes in patient symptoms (i.e., reduction in tremor) (11). Therefore, this step represents a clinically meaningful surgical manipulation.

To test the feasibility of fMRI acquisition and standardize the imaging preprocessing pipeline, we acquired fMRI in essential tremor (ET) patients undergoing asleep VIM DBS. We hypothesized that it was feasible to acquire a reliable and responsive fMRI during asleep VIM DBS surgery. Feasibility was defined as a temporal signal-to-noise ratio (TSNR) >100. Furthermore, we tested whether the fMRI signal was responsive to intraoperative surgical manipulation. We acquired resting-state fMRI before and after the insertion of a ceramic cannula into the brain for this experiment. The insertion of the brain cannula to the surgical target precedes the DBS electrode insertion during asleep DBS to check for anatomical accuracy. This investigation will provide feasibility data for research efforts to develop fMRI-based intraoperative feedback as a response or predictive biomarker to guide surgical decision making during asleep DBS surgery.

## Methods

We prospectively enrolled 10 patients (nine with essential tremor (ET) and one with Parkinson disease with antecedent ET). All patients provided written, informed consent to participate in this institutional review board-approved study (Study ID#2016H0454). The data for this study can be made available through reasonable requests made to the senior author.

### Surgical Procedure

Patients underwent asleep unilateral ventral intermediate thalamic nucleus DBS on a 3-T scanner using the Clearpoint system (4). The surgical target was identified with tractography in patient-specific diffusion imaging acquired preoperatively using a previously described methodology (7). Briefly, we defined the lateral and posterior borders of the VIM by tracking the pyramidal tract and medial lemniscus, respectively. After this step, we created a cubic VIM ROI such that its center was 3 mm medial and anterior to the pyramidal tract and the lemniscus borders. Fiber tracking from this VIM ROI was then performed without using other waypoints. A surgical target was marked at the VIM ROI's inferior border to maximize the passage of the DBS trajectory through the VIM.

### Intraoperative fMRI Acquisition

**Figure 1:**
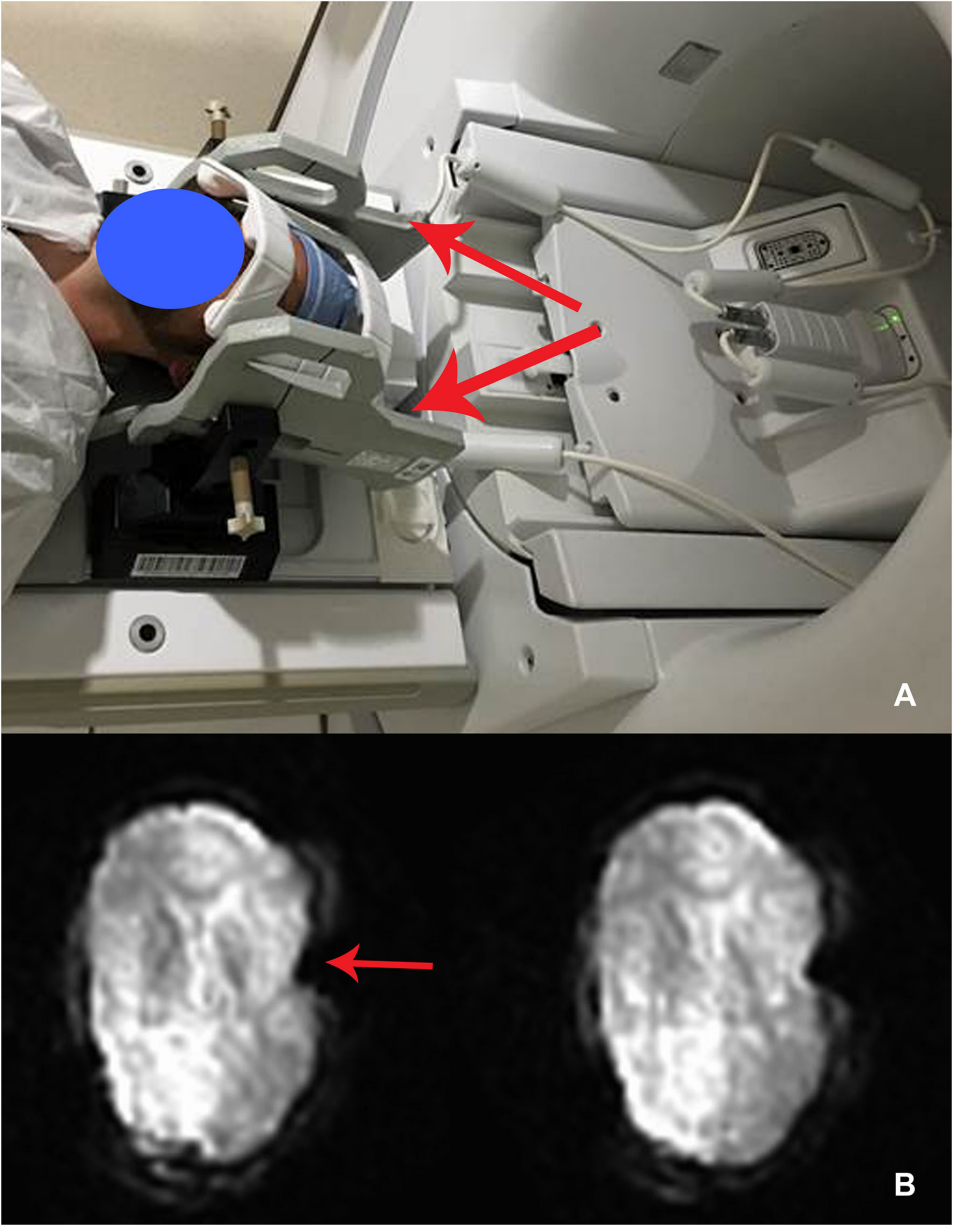
**(A)** Intraoperative set up using receive-only six-channel phase array coil (two red arrows) (left). **(B)** EPI images with the susceptibility artifact due to the metallic pins used to immobilize the head (arrow) (right).

For intraoperative imaging, we developed an *ad-hoc* setup for real-time fMRI acquisition with two six-channel phase array FlexCoils™ (receive only) positioned on both sides of the head, parallel to one another. Two echo-planar (EPI) resting-state fMRI (flip angle = 90°, the field of view = 22 ×22 cm, acquisition matrix = 64 ×64, repetition time = 2,000 ms, 200 runs, voxel size = 3.75 ×3.75 ×3.75 mm^3^) were acquired before and after the insertion of the brain cannula. The setup is outlined in Figure 1A.

### Feasibility of fMRI Acquisition

We calculated the time course stability of fMRI using TSNR, which is computed by dividing the mean of a time series by its standard deviation in a defined brain region. The optimal ranges for TSNR with 3-T scanners were estimated to be between 78 and 90 for gray matter and 110 and 160 for white matter, respectively (12, 13). A temporal signal-to-noise ratio >100 was considered to be indicative of feasibility.

### Connectivity Analysis

Connectivity analysis was performed using the AFNI software package (14) with optimizations to improve pre- to post-cannula insertion image registration and bandpass filtering of multiple frequencies. Standard processing steps were followed (15), and principal component analysis (AFNI 3dPVmap) was used to decompose noise frequencies for regression analysis. We identified seven regions of interest (ROIs) in the tremor network per hemisphere (Eickhoff-Zilles macrolabels) (16–19) and two “control” regions, bilateral middle temporal gyri.

### DBS Contact Localization

The DBS contact locations were derived using the open-source software LEAD-DBS v2.1.7 (http://www.lead-dbs.org). The post-operative CT images were co-registered to the preoperative MRI images and non-linearly coregistered to the MNI ICBM 2009c T1 template. The contacts were localized in the template space using the CT metallic artifact (20).

### Tremor Assessment

The tremor was assessed using a clinical rating scale for tremors, which included assessment included with motor tasks (i.e., action, posture, and intention) and writing assessment (spiral, straight line, and handwriting) (21). The severity was scored between 0 and 4 (0 = no tremor; 4 = severe tremor), and the total scores for the three handwriting specimens were calculated (range 0–20). The percentage tremor improvement was calculated by subtracting the tremor scores at 1 year (±3 months) follow-up from the baseline tremor score.

## Statistical Analysis

Continuous variables were summarized with mean and standard deviation and categorical variables as proportions.

For each subject, we computed the pairwise linear (Pearson) correlation coefficients between ROI, transformed to z-score, and input into the matrix-based-analysis (MBA) software for a Bayesian multilevel (BML) modeling approach to the statistical inference of matrix-based data (22). Briefly, the statistical analysis was performed at the population level MBA with all the ROIs incorporated in one Bayesian multilevel model. We modeled the interaction between imaging time points (before/after cannula insertion) as a categorical variable. A *p*-value <0.05 was considered significant. In contrast to the traditional voxel-wise data analysis, we used this approach because it summarizes the effects of interest within the Bayesian framework *via* posterior distributions without resorting to arbitrary thresholding decisions.

## Results

**Figure 2:**
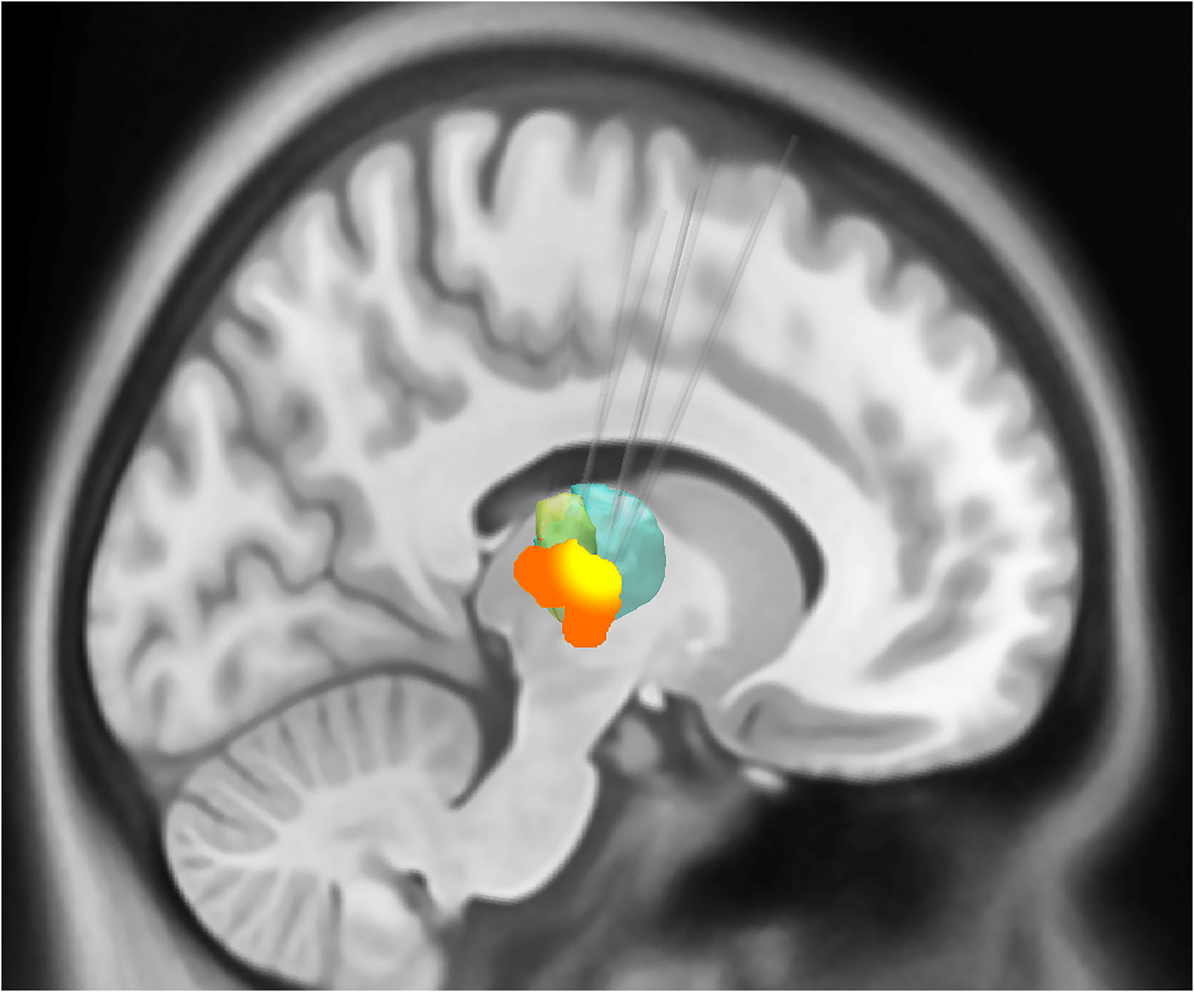
Sagittal representation of the combined volume of stimulation from the active electrodes at the group level overlaid on the motor thalamic atlas region (Oxford thalamic connectivity atlas, Vim represented as green and ventral oralis anterior and posterior nuclei with bottle green). The group-level volumes of activated tissue (VTA) are represented in orange, while a higher overlap (80% or greater) between individual VTAs is shown in yellow color. The VIM target's average distance from the lateral wall of the third ventricle was 10.1 mm (SD 1.55). The target was on an average of 8.83 mm (SD 1.35) anterior to the posterior commissure.

The demographics, anesthesia regimens, and tremor outcomes data are shown in Table 1. It is important to note that the duration of anesthesia as reported in Table 1 is includes induction, frame placement, stage 1 (unilateral DBS electrode insertion), and stage 2 (insertion of battery pack and connection to the intracranial lead), plus extubation. The actual surgical time was around 3 h. The group-level electrode trajectories are shown in Figure 2. All patients received a single DBS electrode in the tractography-defined VIM (Model 3389, Medtronic Inc.).

**Table 1:** Demographics, anesthesia regimen, tremor improvement and DBS settings.

**ID** **(Age/gender)**	**Anesthesia regimen and duration**	**Ventilator settings (rate, FiO_**2**_, end-tidal CO_**2)**_**	**Tremor improvement (%)**	**DBS settings (cathode, anode, frequency, pulse width, and amplitude)**
1(72/F)	Fentanyl IV, propofol, rocuronium 6.5 h	12 breaths/min, 21%, 34%	100	0-, 2+, 135 Hz, 100 μs, 4 mA
2(71/F)	Fentanyl IV, propofol, rocuronium 6 h	10 breaths/min, 22%, 32%	91	1-, Case+, 195 Hz, 80 μs, 3.6 mA
3(52/M)	Fentanyl IV, propofol, rocuronium 6 h	10 breaths/min, 22%, 30%	16	1-, 2+, 160 Hz, 70 μs, 2.5 mA
4(65/M)	Fentanyl IV, propofol, rocuronium 5 h	12 breaths/min, 30%, 21%	66	0-, 3+, 160 Hz, 90 μs, 3.6 mA
5(76/M)	Fentanyl IV, propofol, rocuronium 5 h	12 breaths/min, 24%, 31%	76	0-, 1+, 155 Hz, 110 μs, 3.6 mA
6(77/F)	Propofol, vecuronium 6 h	12 breaths/min, 21%, 34%	66	0-, 2+, 125 Hz, 90 μs, 3.6 mA
7(73/F)	Fentanyl IV, propofol, rocuronium 6 h	12 breaths/min, 21%, 34%	84	0-, Case+, 130 Hz, 120 μs, 2.8 mA
8(79/M)	Fentanyl IV, Propofol, rocuronium 6 h	14 breaths/min, 18%, 27%	83	1-, Case+, 130 Hz, 60 μs, 2.3 mA
9(71/M)	Fentanyl IV, propofol, rocuronium 5 h	13 breaths/min, 21%, 36%	83	0-, 3+, 180 Hz, 90 μs, 7.9 mA
10(73/F)	Fentanyl IV, propofol, rocuronium 6 h	10 breaths/min, 32%, 22%	85	1-, 3+, 180 Hz, 90 μs, 4.5 mA

We were able to acquire fMRI in all 10 patients. The fMRI acquisition was only 10 min duration, and we acquired two runs of it with total 20 min of additional scanning time, compared with standard of care. The average temporal SNR was 190.23 (SD 68.99), with no significant difference between pre- and post-cannula insertion (*p* = 0.2) conditions. The motion variable analysis did not show motion above 1 mm (mean 0.5; SD 0.2) due to head immobilization. The immobilization pins induced a significant inhomogeneity artifact in the EPI images (Figure 1B). We corrected this artifact by affinely aligning the two runs, pre- and post-cannula insertion, before registering both to the anatomical images.

We observed a low-frequency (~every 100 s) and a high-frequency oscillatory BOLD signal artifact (Supplementary Figure 1) in four patients—these artifacts, where present, were specifically included in the general linear model (GLM) together with the motion parameters. Secondly, we extracted the artifact frequency by computing the first two principal component vectors of each dataset (3dPVmap software—AfNI) and used them for targeted bandpassing during preprocessing.

**Figure 3:**
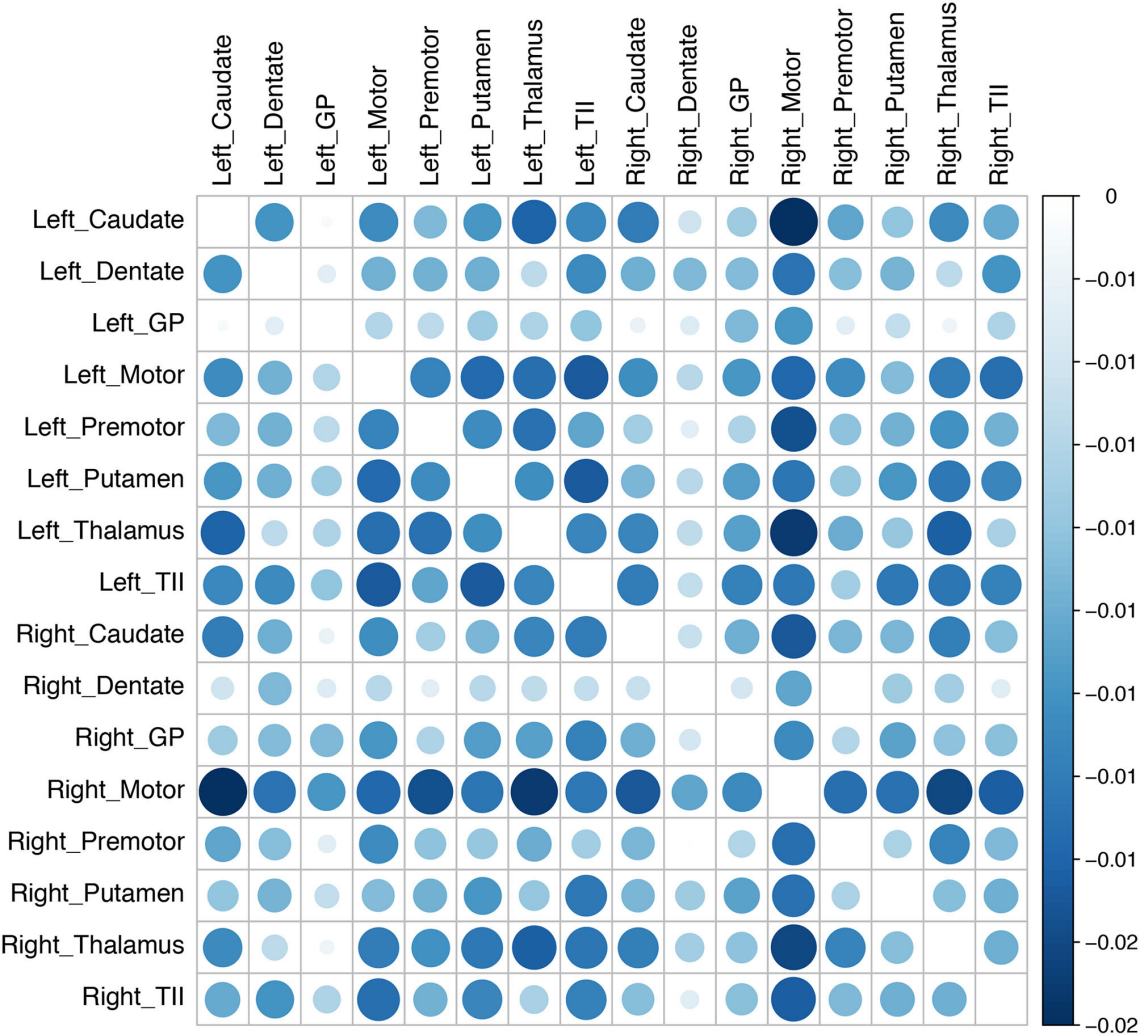
Changes in connectivity in the tremor network after brain cannula insertion. The changes in connectivity are color coded in the matrix (blue implies decline and red implies increase). The magnitude of connectivity change is indicated by the bubble size, while the color intensity represents the statistical significance. There was an overall decline in the tremor network's connectivity after the canula insertion, although the group-level results did not reach statistical significance (GP, globus pallidus; TII, middle temporal gyrus).

After cannula insertion, there was a generalized decrease in connectivity between the left motor and the left premotor cortex, left putamen, left thalamus, right motor cortex, right premotor cortex, and right thalamus. Similarly, the connectivity of the right motor cortex with all the other ROIs in the tremor network also decreased. The probability interval (uncertainty) of the change was not statistically significant (Figure 3).

## Discussion

Here, we show the feasibility of acquiring a reliable and responsive fMRI in patients undergoing asleep VIM DBS. We observed two notable artifacts and developed and released an analysis pipeline to correct for distortions during intraoperative imaging. Processing options to address artifacts such as these are now included as new options for afni_proc.py in the AFNI software package.

Conventionally, the treatment of medication-refractory ET with DBS is performed with the patient “awake,” using microelectrode recordings and test stimulation (23). Despite its excellent long-term effectiveness (24), some patients cannot undergo awake DBS surgery either because of high surgical risk or unwillingness to stay awake due to anxiety and fear. With the enhanced accessibility of intraoperative imaging, asleep DBS surgery has recently gained popularity, and it is reported to be safe and accurate (25, 26). However, asleep VIM DBS remains challenging because this nucleus is not readily visible on conventional structural imaging. Furthermore, physiological feedback during asleep VIM DBS is currently lacking, and it may ultimately be required as a surrogate for long-term clinical outcomes similar to intraoperative testing during awake DBS (27).

As a first step toward this goal, we tested the feasibility of acquiring fMRI during asleep DBS. We found fMRI acquisition feasible but associated with two artifacts: a susceptibility artifact and an oscillatory artifact. The susceptibility artifact was related to metallic pins used for head immobilization during surgery and can be addressed using non-metallic pins. However, the exact origin of these oscillatory artifacts remains uncertain. These artifacts could be intrinsic to the setup or associated with external factors (e.g., the ventilator could induce such low-frequency signals). Traditionally BOLD fMRI suffers from numerous sources of structured noise (28), which are difficult to isolate and affect the fMRI signal's quality. These include rapid and slow head movements, physiological activity (breathing and heart-beat), and scanner artifacts. Even after conventional preprocessing steps, such as slice-timing correction, motion correction, high-pass filtering, and spatial smoothing, some of these artifacts still remain (29) in any fMRI analysis. In particular, clinical fMRI has practical constraints that make the elimination of such artifacts challenging and introduce additional artifacts (e.g., *via* oxygen delivery or health monitoring equipment). Therefore, one must attempt to reduce these artifacts during early testing of the clinical or intraoperative fMRI analysis paradigm. As with all scanning centers, gauging the scans' consistency over time, such as scanning phantoms or volunteers at regular intervals, is also strongly advised.

In the scanning center used in the present work, efforts are underway to determine the root causes of the artifacts described above, including testing with a new FlexCoils™ coil and monitoring for specific noise sources, including from the ventilator. Nierhaus et al. (30) reported an EEG artifact during simultaneous MRI acquisition in a Siemens scanner with frequency peaks in the range of physiologically relevant brain rhythms (gamma frequency range) due to the MRI ventilation system. A similar issue to our knowledge has not been reported in the fMRI literature. The artifacts we observed are likely related, in part, to some of the clinical scan setup and monitoring. In our view, these represent a practical concern of note for intraoperative scanning and something the researchers should be mindful about.

We noticed that the fMRI signal was responsive to the brain cannula insertion, and connectivity analysis showed an overall decline in connectivity in the tremor network, especially the connectivity between the bilateral motor cortices and other nodes in the tremor network. Nevertheless, group analysis failed to reach statistical significance. The insertion of a brain cannula represents a clinically meaningful surgical manipulation since patients often experience immediate, although transient, tremor reduction after DBS placement (31, 32). Notably, general anesthetics also reduce the global cerebral metabolic rate, thereby reducing the long-range functional connectivity. The necessity of scanning subjects with anesthesia is another practical limitation of intraoperative scanning, compared with research paradigms. At the anesthetic depth characterized by the subjects' unresponsiveness, a partial—but not complete—reduction in connectivity is generally observed (33), up to almost 25% lower under general anesthesia than in an awake state (34). Standardizing the anesthetic regimen and using drug dosages similar to those used during intraoperative physiological monitoring can reduce variability in fMRI signal attributed to the anesthetic depth. We standardized the anesthesia regimen for all the cases based on a slow injection of 0.5 mg/kg of Propofol over 3 to 5 min followed immediately by a maintenance infusion at the lowest rate compatible with the maintenance of sedation. Carbon dioxide concentrations were maintained within the normal physiological levels of between 4 and 5.7 kilopascals (kPa), with a respiratory rate of 10–15 breaths per minute, and blood oxygen saturations were maintained >95% with the use of increased inspired oxygen concentration as it is standard clinical practice.

The determination of stimulation-evoked fMRI changes will be an exciting next step since therapeutic DBS was shown to induce network-specific fMRI changes across subjects (35, 36). The methodology and analysis pipeline developed through this investigation can be deployed in future studies to develop stimulation-induced intraoperative physiological feedback during asleep DBS surgery. These efforts are limited by the manufacturer's guidelines prohibiting MRI acquisition with 3 T and indwelling DBS hardware (37). Continued investigations to determine the safety conditions for scanning with DBS hardware will enable further testing of stimulation-induced fMRI changes.

In this paper, we purposefully report long-term tremor outcomes after asleep DBS surgery in accordance to the current practice in our field [see, for example, Gravbrot et al. (38)].

## Conclusions

It was feasible to acquire functional MRI during neurosurgery under general anesthesia. In a similar intraoperative setting, specific sources of artifacts (e.g., ventilation, metal pins, frame, general anesthesia) need to be accounted for when applying the standard fMRI preprocessing steps to ensure the robustness of the data. The suggested acquisition steps and the preprocessing pipeline will help future investigators to determine functional connectivity changes during asleep DBS and develop response biomarkers suitable for live functional MRI feedback to guide surgical decision making.

## Data Availability Statement

The raw data supporting the conclusions of this article will be made available by the authors, without undue reservation.

## Ethics Statement

The studies involving human participants were reviewed and approved by Buckeye IRB-The Ohio State University. The patients/participants provided their written informed consent to participate in this study.

## Author's Note

Portions of this work were presented as an oral presentation at the World Society of Stereotactic and Functional Neurosurgery Meeting, New York City, NY, June 27, 2019.

## Author Contributions

FS and VK: conception and design of the study, acquisition and analysis of data, and drafting a significant portion of the manuscript or figures. PT, RR, GC, and DG: acquisition and analysis of data. All authors contributed to the article and approved the submitted version.

## Conflict of Interest

The authors declare that the research was conducted in the absence of any commercial or financial relationships that could be construed as a potential conflict of interest.

## Supplementary Material

Supplementary Figure 1 An example of high- (enorm and dP plots) and low-frequency (outlier and dl plots) artifacts were noted during the post-processing in one of the four patients. The AFNI quality check output is shown.

Supplementary Figure 2 An example of the regions of interest (ROI) used to perform the connectivity analysis that is shown in Figure 3.
