# Supplementary figures and images for: Functional Neuroimaging During Asleep DBS Surgery: A Proof of Concept Study
Source: Front Neurol. 2021 Jun 28;12:659002. doi: 10.3389/fneur.2021.659002 (PMC8273165; doi:10.3389/fneur.2021.659002)

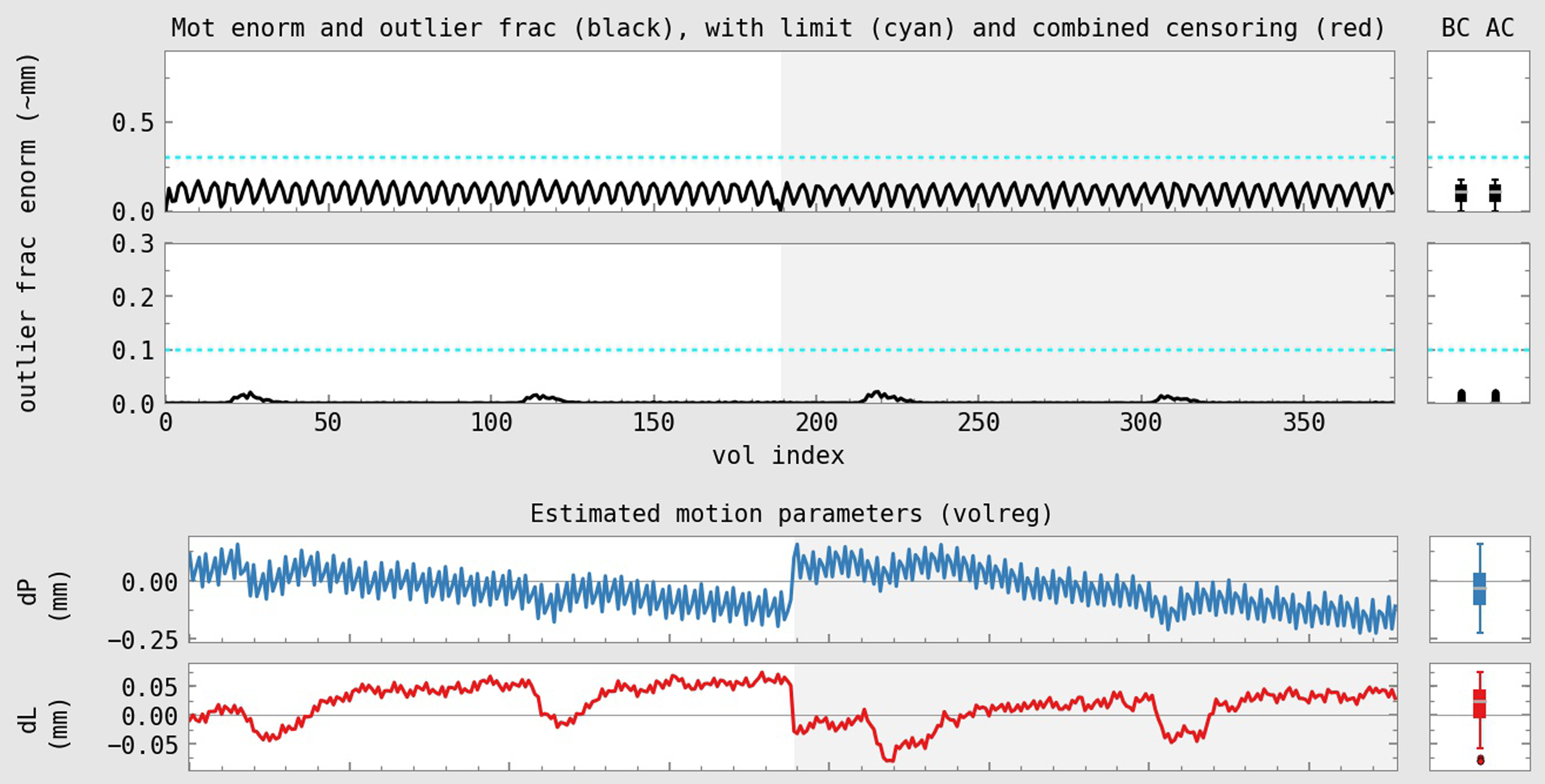

Supplement: Supplementary Figure 1 — An example of high- (enorm and dP plots) and low-frequency (outlier and dl plots) artifacts were noted during the post-processing in one of the four patients. The AFNI quality check output is shown. [file Image_1.TIF]

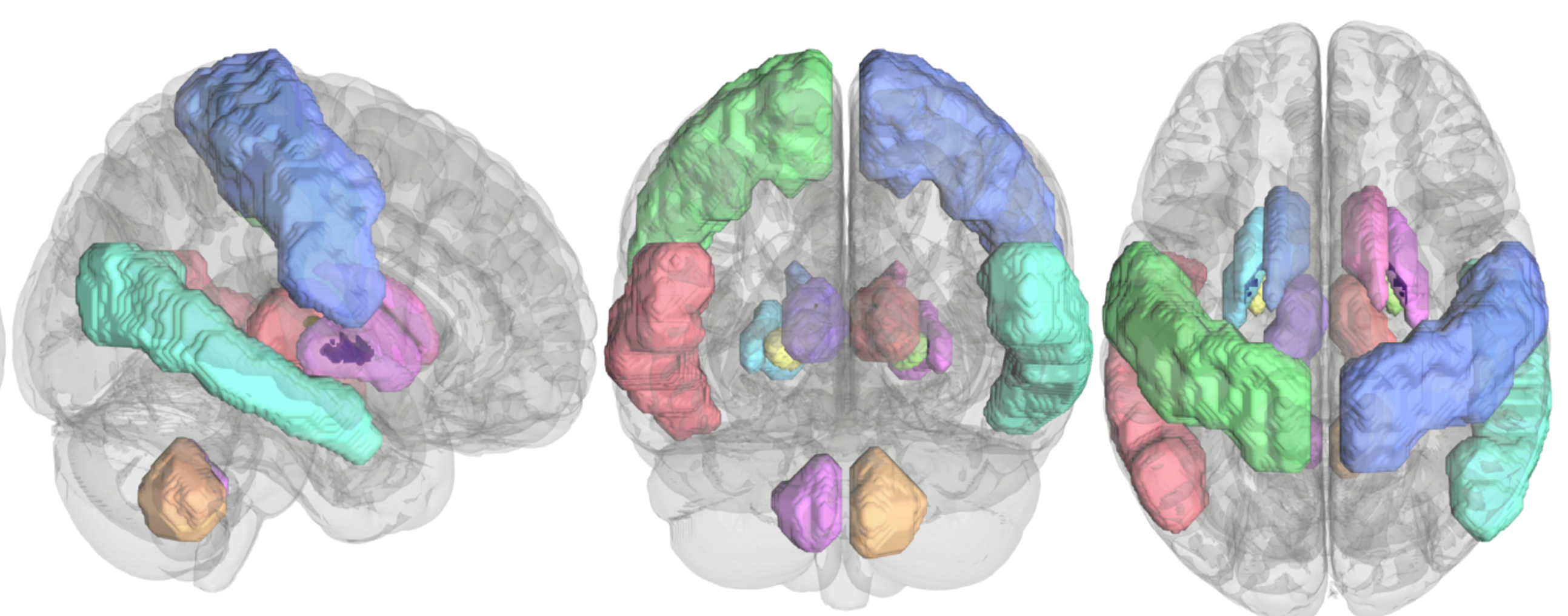

Supplement: Supplementary Figure 2 — An example of the regions of interest (ROI) used to perform the connectivity analysis that is shown in Figure 3. [file Image_2.TIFF]
